# Detection and identification of spotted fever group Rickettsiae and Ehrlichiae in African ticks.

**DOI:** 10.3201/eid0706.010616

**Published:** 2001

**Authors:** P Parola, H Inokuma, J L Camicas, P Brouqui, D Raoult

**Affiliations:** Unité des Rickettsies, CNRS UMR 6020, Marseille, France.

## Abstract

Rickettsia africae, a recently identified pathogen, was detected for the first time in Amblyomma ticks from Niger, Mali, Burundi, and Sudan, and "R. mongolotimonae" was identified for the first time in Africa. Rickettsiae of unknown pathogenicity and two new ehrlichiae of the Ehrlichia canis group were identified in ticks from Mali and Niger.


### Detection and Identification of Spotted Fever Group Rickettsiae and *Ehrlichiae* in African Ticks

**Philippe Parola,* Hisashi Inokuma,* Jean-Louis Camicas,†
Philippe Brouqui,* and Didier Raoult***
*Unité des Rickettsies, CNRS UMR 6020, Marseille, France;
and †Centre IRD, Montpellier, France

*Rickettsia africae*, a recently identified pathogen, was detected for the first time in *Amblyomma* ticks from Niger, Mali, Burundi, and Sudan, and "*R. mongolotimonae*" was identified for the first time in Africa. Rickettsiae of unknown pathogenicity and two new *Ehrlichiae* of the *Ehrlichia canis* group were identified in ticks from Mali and Niger.

Spotted fever group Rickettsiae and *Ehrlichiae* are obligate intracellular gram-negative bacteria associated with arthropods, mainly ticks. While feeding, ticks can transmit these microorganisms to humans and animals (1). Two human tick-borne rickettsioses are known to occur in Africa (2). Mediterranean spotted fever, caused by *Rickettsia conorii*, is transmitted by the brown dog tick, *Rhipicephalus sanguineus*, which is well adapted to urban environments. *R. conorii* is prevalent in the Mediterranean area (Tunisia, Algeria, Morocco, Libya, and Egypt) and has also been isolated or detected in Kenya, Central Africa, Zimbabwe, and South Africa (2). Although African tick bite fever has been recognized since the beginning of the century as a rural disease usually contracted from ticks of cattle and game, it was regarded as synonymous with Mediterranean spotted fever, until the first human infection with *R. africae* was reported from Zimbabwe in 1992. Subsequently, numerous cases have been reported in tourists returning from southern Africa, where the cattle tick *Amblyomma hebraeum* is the vector (2,3). *R. africae* has also been recovered from *A. variegatum* ticks in Ethiopia and central Africa (2). In 1992, a survey for antibodies against *Ehrlichia chaffeensis* (the agent of human monocytic ehrlichiosis) in human sera from eight African countries indicated that human ehrlichioses might occur on the continent (4), and subsequently a case (diagnosed by serology only) was reported from Mali (5). Recently, new molecular methods have enabled the development of useful, sensitive, and rapid tools to detect and identify tick-borne pathogens in arthropods, including ticks (6). In this work, we tested ticks from Africa for rickettsial and ehrlichial DNA using polymerase chain reaction (PCR) and sequence analysis of amplified products.

### Materials and Methods

Ticks were kept frozen at -20°C (in Niger) or at -80°C (in other countries) before being tested. DNA of each tick was extracted as described (7). Rickettsial and ehrlichial DNA was detected by PCR as described, using specific primers (Table).

Address for correspondence: Didier Raoult, Unité des Rickettsies, Faculté de Médecine, Université de la Méditerranée, CNRS UMR 6020, 27 Bd Jean Moulin, 13385 Marseille Cedex 5, France; fax: 33-491-83-0390; e-mail: Didier.Raoult@medecine.univ-mrs.fr.

The sequences of PCR products were obtained and analyzed with the corresponding sequences of rickettsial or ehrlichial species as described (7). Multiple alignment analysis was performed by using the ClustalW program version 1.8 in the DNA Data Bank of Japan (DDBJ; Mishima, Japan [http://www.ddbj.nig.ac.jp/htmls/E-mail/clustalw-e.html]). All sequences used in the study are available in GenBank; the accession numbers of the new genotypes detected in this work are shown in the Table footnotes.

### Results

Rickettsial DNA was detected in 24 (7.2%) of the 332 ticks examined (Table). *R. africae* was detected from *A. variegatum* from Mali (2/6), Niger (2/6), and Burundi (1/13), and from 1 of 16 *A. lepidum* from the Sudan. *R. aeschlimmanii* was detected in *Hyalomma marginatum rufipes* from Niger and Mali (8/24 and 3/20, respectively) and *R. massiliae* in 2/37 *Rh. muhsamae* from Mali. Further, three new *ompA* sequences (590 bp) were obtained from *A. variegatum* from Mali and Niger (Table). These were 99.3%–99.5% identical to those of *R. africae*. In the phylogenetic tree based on these *ompA* sequences, the three rickettsiae (named RAv1, RAv3, RAv9) were closely related to one another (95.7% bootstrap value) and branched with *R. africae* (86.1% bootstrap value) (data not shown). Partial sequences (316 bp) of the *gltA* gene of RAv1, RAv3, and RAv9 were also found to be closely related to those of *R. africae* (99% of similarity). Two new 16S rRNA ehrlichial genotypes were detected, including ERm58 (1,380 bp) in 7/37 *Rh. muhsamae* from Mali and EHt224 (1366 bp) in 1/5 *H. truncatum* from Niger. Both sequences were very similar (99.34% similarity), but different from those described for all the known ehrlichiae (i.e., 98.55% similarity with *E. chaffeensis*, 98.26% with *E. canis* and *E. ewingii*, and 97.75% with *E. muris* and *Cowdria ruminantium*). In a phylogenetic tree based on 16S rRNA gene sequences, ERm58 and EHt224 were found to be closely related and to belong to the *E. canis* group (data not shown). Enlarged *gltA* sequences of ERm58 (1,140 bp) and EHt224 (1,189 bp) were also obtained from the above ticks. Phylogenetic analyses of these sequences confirmed that ERm58 and EHt224 belonged to the *E. canis* group (data not shown).

